# Establishment and validation of a nomogram to predict thirty-day unplanned reoperations of primary anastomosis in neonates with intestinal atresias

**DOI:** 10.3389/fped.2025.1660827

**Published:** 2025-10-29

**Authors:** Zhixiong Lin, Weiming Chen, Zhihao Fang, Fei Chen, Yifan Fang, Mingkun Liu

**Affiliations:** ^1^ Department of Pediatric Surgery, Fujian Children’s Hospital (Fujian Branch of Shanghai Children’s Medical Center), College of Clinical Medicine for Obstetrics & Gynecology and Pediatrics, Fujian Medical University, Fuzhou, Fujian, China; ^2^ Department of Pediatric Surgery, Fujian Maternity and Child Health Hospital, College of Clinical Medicine for Obstetrics & Gynecology and Pediatrics, Fujian Medical University, Fuzhou, Fujian, China; ^3^ Department of Operating Room, Fujian Children’s Hospital (Fujian Branch of Shanghai Children’s Medical Center), College of Clinical Medicine for Obstetrics & Gynecology and Pediatrics, Fujian Medical University, Fuzhou, Fujian, China

**Keywords:** intestinal atresia, reoperation, nomogram, risk factor, neonate

## Abstract

**Background/purpose:**

Unplanned reoperation rates becoming a critical metric for evaluating healthcare quality and have received increasing attention in recent years. Intestinal atresia (IA) has a high rate of unplanned reoperations. The purpose of this study is to evaluate the thirty-day unplanned reoperation rates and their risk factors in neonates with intestinal atresias after primary anastomosis surgery, and to construct a predictive nomogram.

**Methods:**

We developed and internally validated a predictive model from a retrospective cohort of 200 neonates admitted to our hospital for primary anastomosis surgery. The primary outcome was thirty-day unplanned reoperation rates. Independent factors significantly associated with thirty-day unplanned reoperation rates were identified using multivariable logistic regression analysis. The effectiveness of the developed nomogram was evaluated through calibration, discrimination, and clinical utility.

**Results:**

The incidence of thirty-day unplanned reoperation rates was 11%. Multivariable analysis identified the type of bowel anastomosis and combined meconium peritonitis as independent factors predicting thirty-day unplanned reoperation rates. The derivation model showed good discrimination, with a C-index of 0.791 (95% CI, 0.685–0.897), and good calibration (Hosmer–Lemeshow test *P* = 0.231). The analysis of the decision curve showed that the nomogram was beneficial in clinical practice.

**Conclusion:**

We developed a nomogram to predict thirty-day unplanned reoperations of primary anastomosis in neonates with IA. This prediction model may enable assist in clinical decision-making, patient counseling, and treatment planning.

## Introduction

Intestinal atresia (IA) as one of the most common causes of neonatal bowel obstruction requires operative repair in the early neonatal period (1). Primary anastomosis has emerged as the preferred surgical approach due to its advantages of shorter hospital stays and avoidance of stoma-related complications (2–4). The unplanned reoperations rates in IA varied considerably from 11%–25% (5–7). Unplanned reoperation serves as a crucial indicator of surgical quality for evaluating a hospital's capability to deliver safe and efficient care (8). Reoperations often associated with prolonged hospital stays and increased healthcare costs (9). Unfortunately, there still exists a scarcity of studies about the incidence of unplanned reoperation after primary anastomosis in IA neonates.

Identifying risk factors for unplanned reoperations could make a significant contributions to enhancing surgery quality, thus, the objective of this study lies in the investigation of risk factors underlying the aforementioned issue. To this end, we developed and validated a comprehensive prediction model, aiming to optimize clinical decision-making and improve outcomes for these patients.

## Materials and methods

**Figure 1:**
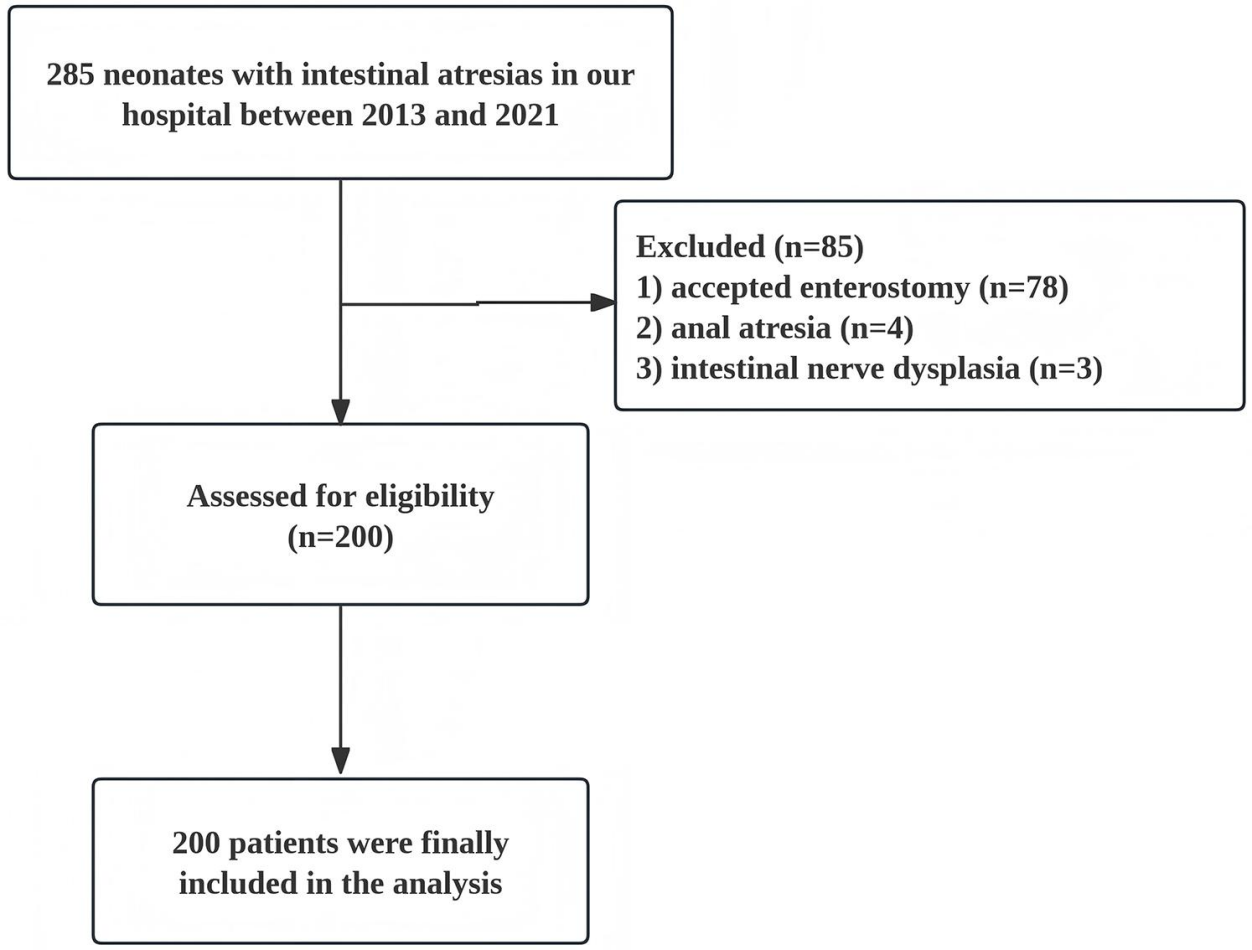
Flow chart of the study cohort.

In this study, we conducted a retrospective analysis of clinical data from 200 neonates with IA who underwent primary anastomosis surgery at Fujian Maternity and Child Health Hospital between May 2013 and May 2021. The study include neonatal patients with intestinal atresia in the jejunum and ileum who received primary anastomosis; while patients who accepted enterostomy or suffered from anal atresia, concomitant intestinal nerve dysplasia, and other serious congenital malformations were excluded (Figure 1). The study began after approval by the ethics committee at Fujian Maternity and Child Health Hospital (No. 2024KY096).

The analyzed data encompassed the following parameters: patient's week of gestation, prematurity, birth weight, prenatal ultrasound, age at surgery, site and type of intestinal atresia, type of anastomosis, coexistence of meconium peritonitis (MP), concomitant malformations, intestinal necrosis/perforation, and whether reoperation was performed or not. Thirty-day unplanned reoperation can be defined as a surgical intervention required due to the complication arising from primary procedure within 30 days postoperation.

## Statistical analysis

Statistical software IBM SPSS Statistics 26 and R version-4.2.0 (https://www.r-project.org) was used for data analysis. Quantitative data conforming to normal distribution was represented by *x* *±* *s*, quantitative data not conforming to normal distribution was represented by *M (P25,P75)*, and comparison between two groups was performed by independent sample *t* test or non-parametric test. Qualitative data were presented in percentage *n* (%); chi-square test or Fisher test were used for comparison between groups. The selected relevant factors with *p* value < 0.05 were further analyzed by one-way and multi-factor binary logistics regression analysis, and Statistical significance was defined as *p* value < 0.05.

The discriminatory ability of the developed nomogram was assessed using the C-index and ROC curve analysis. The C-index statistic ranges from 0.5 (no discrimination) to 1.0 (perfect discrimination). The calibration curves were applied for examine the agreement between predicted and observed probabilities. Subsequently, the clinical utility of the nomogram was evaluated through decision curve analysis (DCA).

## Results

### Baseline characteristics

Among 200 patients included into the study, 112 were male and 88 were female, with an average weight of 2,946 ± 562 g. Among them, 13 (6.5%) patients were premature and 104 (52%) had abnormal prenatal ultrasound findings. In terms of intestinal atresia classification, type I occurred in 78 cases (39%), type II in 32 cases (16%), type III in 58 cases (29%), and type IV in 32 cases (16%). Regarding the site of intestinal atresia, duodenal atresia accounted for 45 cases (22.50%), jejunal atresia for 44 cases (22.00%), and ileal atresia for 111 cases (55.50%). For the method of intestinal anastomosis, 113 cases (56.50%) used end-to-side oblique anastomosis, 53 cases (26.50%) underwent septum resection, and 34 cases (17.00%) adopted other anastomosis methods, including end-to-end, side-to-side, and diamond-shaped anastomoses. During the surgery, it was found that 60 cases (30%) were complicated with MP, and 20 cases (10%) exhibited intestinal perforation and necrosis discovered intraoperatively. Of all the patients included in the study, a total of 22 experienced unplanned reoperations within 30 days post-surgery, resulting in an unplanned reoperation rate of 11%. Table 1 presents the baseline characteristics of the patients diagnosed with IA and received primary anastomosis surgery.

**Table 1:** Demographic characteristics of 200 IA patients.

Variables		Total (*n* = 200)	No Reoperation (*n* = 178)	Reoperatio*n* (*n* = 88)	*χ^2^/Z/t*	*P*
Sex, *n* (%)	Female	88(44.00)	80	8	0.585	0.444
	Male	112 (56.00)	98	14		
Age, *n* (%)	<2 d	106 (53.00)	96	10	−2.117	0.034
	2–5 d	55 (27.50)	38	1		
	≥5 d	39 (19.50)	28	11		
Fetal ultrasound, *n* (%)	No	96 (48.00)	81	15	3.939	0.047
	Yes	104 (52.00)	96	7		
Prematurity, *n* (%)	No	187 (93.50)	167	20	0.273	0.640
	Yes	13 (6.50)	11	2		
Stage, *n* (%)	Ⅰ	78 (39.00)	75	3	−2.655	0.008
	II	32 (16.00)	28	4		
	III	58 (29.00)	49	9		
	IV	32 (16.00)	26	6		
Site, *n* (%)	Duodenum	45 (22.50)	43	2	−1.510	0.131
	Jejunum	44 (22.00)	39	5		
	Ileum	111 (55.50)	96	15		
Anastomotic method, *n *(%)	End-to-side	113 (56.50)	103	10	−2.265	0.024
	Membrane Resection	53 (26.50)	52	1		
	Others	34 (17.00)	23	11		
Meconium peritonitis, *n* (%)	No	140 (70.00)	131	9	9.962	0.002
	Yes	60 (30.00)	47	13		
Malformation, *n *(%)	No	116 (58.00)	102	14	0.322	0.570
	Yes	84 (42.00)	76	8		
Intestinal necrosis perforation, *n *(%)	No	180 (90.00)	160	20	0.023	1.000
	Yes	20 (10.00)	18	2		
Weight quantile, *n *(%)	<2,600 g	50 (25.00)	44	6	−0.210	0.834
	2,600–<3,000 g	48 (24.00)	45	3		
	3,000–≤3,300 g	54 (27.00)	46	8		
	>3,300 g	48 (24.00)	43	5		

### Risk factors for thirty-day unplanned reoperations

The results of the univariate analysis indicated that the incidence of thirty-day unplanned reoperations for primary anastomosis in neonates with IA can be influenced by factors such as age at surgery, fetal ultrasound, stage of intestinal atresia, type of bowel anastomosis and combined with MP (all *P value* < 0.1). In multivariable analysis, risk factors significantly associated with unplanned reoperations were the type of bowel anastomosis and combined with MP (*P* < 0. 05) (Table 2).

**Table 2:** Univariate and multivariate Cox regression analysis of risk factors for 30-day unplanned reoperations.

Variables	Univariate analysis					Multivariate analysis				
	β	S.E	Z	*P*	OR (95% CI)	β	S.E	Z	*P*	OR (95% CI)
Sex										
Female					1.00 (Reference)					
Male	−0.27	0.45	−0.60	0.549	0.76 (0.31–1.85)					
Age										
<2 d					1.00 (Reference)					1.00 (Reference)
2–5 d	0.88	0.47	1.85	0.064*	2.40 (0.95–6.07)	0.96	0.58	1.66	0.097	2.60 (0.84–8.04)
≥5 d	−1.38	1.07	−1.29	0.197	0.25 (0.03–2.04)	−0.61	1.17	−0.52	0.602	0.54 (0.05–5.41)
Fetal ultrasound										
No					1.00 (Reference)					1.00 (Reference)
Yes	−0.94	0.48	−1.96	0.050*	0.39 (0.15–1.00)	−0.93	0.57	−1.65	0.099	0.39 (0.13–1.19)
Prematurity										
No					1.00 (Reference)					
Yes	0.42	0.80	0.52	0.640	1.52 (0.31–7.34)					
Stage										
Ⅰ					1.00 (Reference)					1.00 (Reference)
Ⅱ	1.27	0.80	1.60	0.109	3.57 (0.75–16.97)	0.48	0.95	0.50	0.614	1.61 (0.25–10.40)
Ⅲ	1.52	0.69	2.20	0.028*	4.59 (1.18–17.81)	0.65	0.84	0.77	0.444	1.91 (0.36–10.00)
Ⅳ	1.75	0.74	2.36	0.018*	5.77 (1.35–24.74)	1.15	0.91	1.26	0.206	3.17 (0.53–19.02)
Site										
Duodenum					1.00 (Reference)					
Ileum	1.21	0.77	1.56	0.118	3.36 (0.74–15.34)					
Jejunum	1.01	0.87	1.17	0.241	2.76 (0.51–15.03)					
Anastomotic										
End-to-side					1.00 (Reference)					1.00 (Reference)
Membrane resection	−1.62	1.06	−1.52	0.128	0.20 (0.02–1.59)	−0.03	1.27	−0.02	0.983	0.97 (0.08–11.69)
Others	1.59	0.49	3.23	0.001*	4.93 (1.87–12.97)	1.85	0.56	3.31	<.001	6.34 (2.12–18.92)
Meconium peritonitis										
No					1.00 (Reference)					1.00 (Reference)
Yes	1.39	0.47	2.99	0.003*	4.03 (1.62–10.03)	1.19	0.57	2.09	0.036	3.29 (1.08–10.04)
Malformation										
No					1.00 (Reference)					
Yes	−0.27	0.47	−0.57	0.571	0.77 (0.31–1.92)					
Intestinal necrosis perforation										
No					1.00 (Reference)					
Yes	−0.12	0.78	−0.15	0.880	0.89 (0.19–4.12)					
Weight quantile										
1					1.00 (Reference)					
2	−0.72	0.74	−0.97	0.332	0.49 (0.12–2.08)					
3	0.24	0.58	0.42	0.675	1.28 (0.41–3.97)					
4	−0.16	0.64	−0.25	0.804	0.85 (0.24–3.00)					

OR, odds ratio; CI, confidence interval.

* Variables with *p* < 0.1 in univariate analysis were included in multivariate regression analysis.

### Nomogram visualization and performance

**Figure 2:**
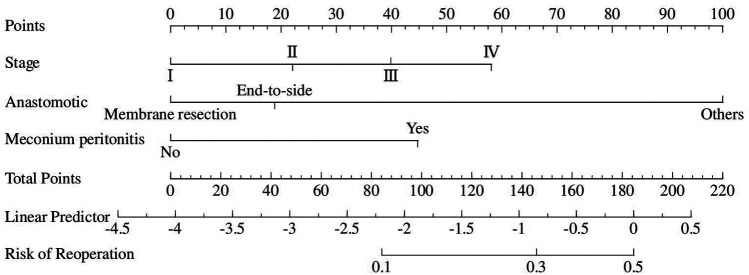
Nomogram predicting the risk of thirty-day unplanned reoperations based on independent risk factors identified from multivariate logistic regression analysis.

**Figure 3:**
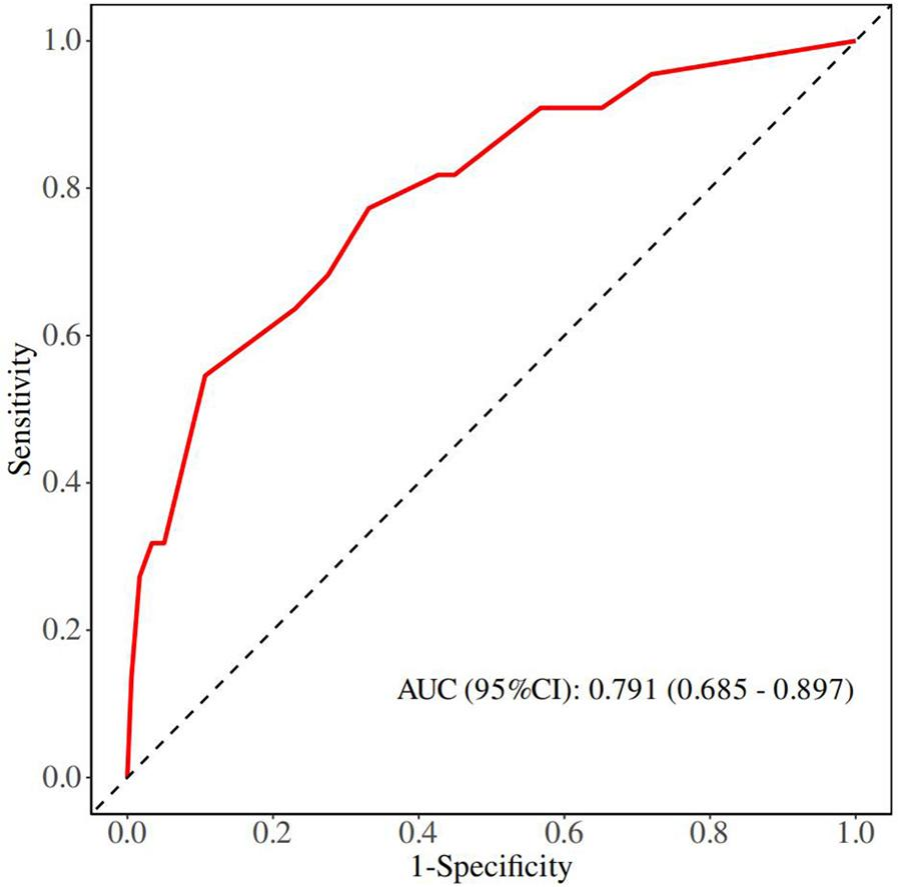
ROC curves of multivariate logistic regression model.

**Figure 4:**
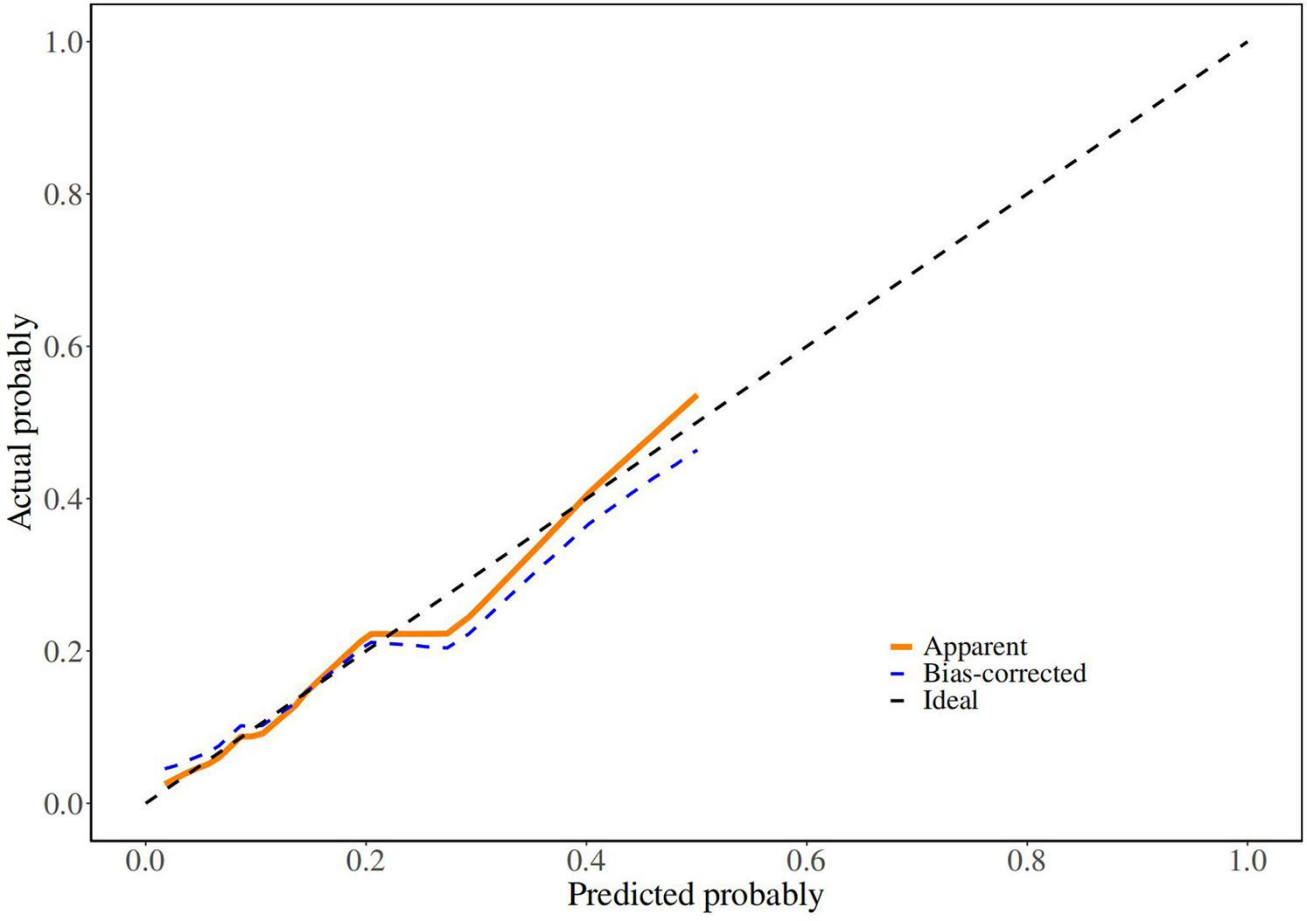
Calibration curve of derivation cohorts and internal bootstrap validation cohorts. In **(B)**, X-axis is the nomogram-predicted probability; Y-axis is the actually observed probability.

Although there was no statistically significant difference in bowel atresia type in the multifactorial analysis, it was included in the model construction since its importance as a clinical variable in bowel atresia disease. The classification of intestinal atresia directly reflects the severity of the intestinal malformation and the complexity of the anatomical defects. These factors jointly influence the difficulty of the surgery, the possibility of postoperative recovery, and the risk of complications. Especially in type IIIb or IV IA, it associated with longer PN support and secondary procedures for intestinal failure (10, 11). Alessandro et al. research shown that sequelae are correlated with the type of atresia and length of residual bowel (12). A predictive nomogram for unplanned reoperations in IA patients after primary anastomosis was developed through incorporating the three risk factors identified from multivariate logistic regression (Figure 2). Type of bowel anastomosis possessed the highest predictive effect, followed by stage of atresia, and MP. The probability of unplanned reoperations can be easily estimated by adding up the scores for each specific variable. Figure 3 showed the the area under the receiver operating characteristic (AUROC) of the predictive nomogram was 0.791 (95% CI, 0.685–0.897) using bootstrapping with 1,000 replicates, as he Harrell C-Index was 0.791. The derivation model presented good calibration, as the *P* value for the Hosmer-Lemeshow test was 0.231. Furthermore, internal validation demonstrated a good calibration comparable to the derivation model, as presented in Figure 4.

### Clinical application

**Figure 5:**
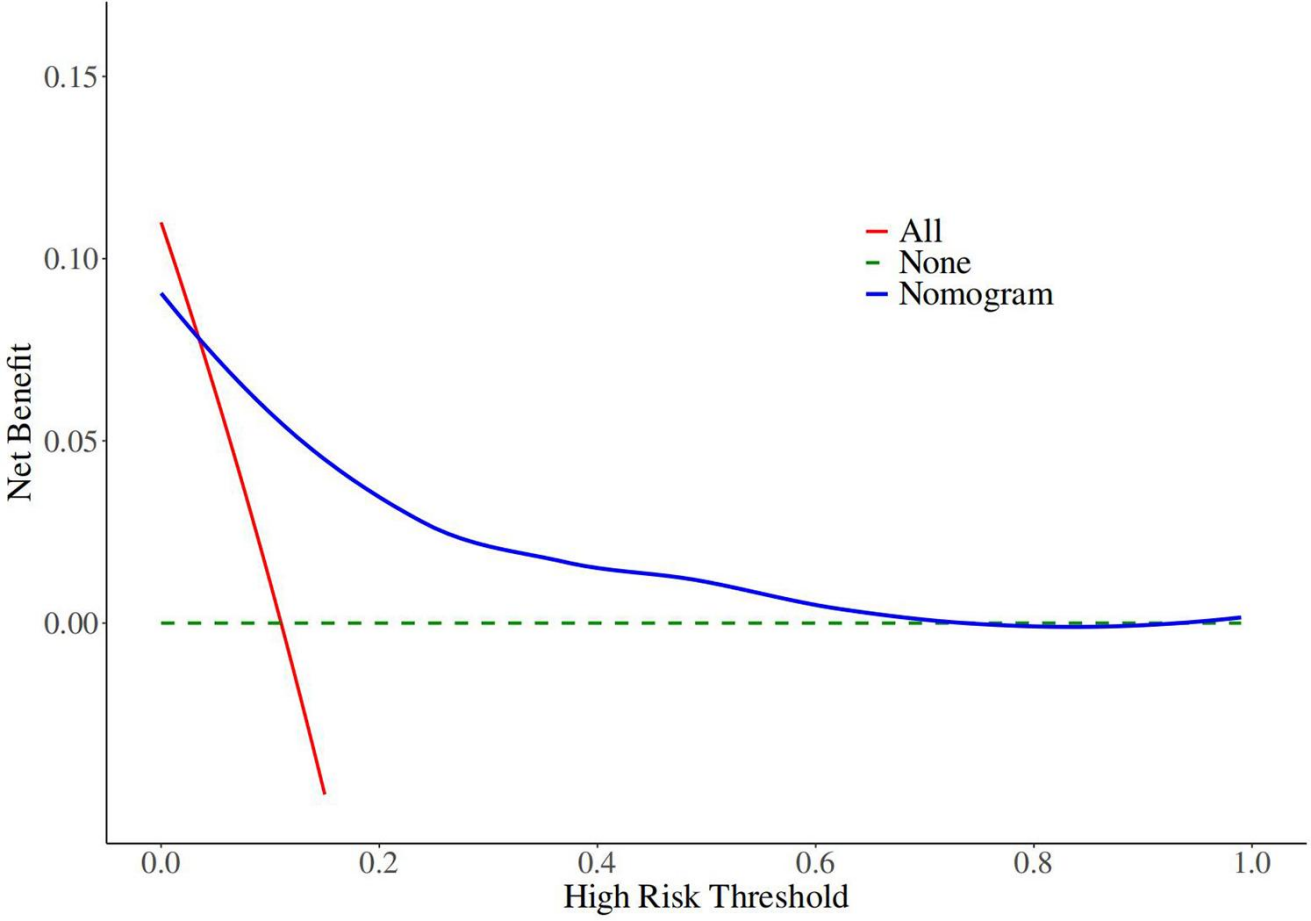
Decision curve analyses for the nomogram.

The decision curves of the nomogram revealed robust clinical utility and substantial net benefit within the risk range of 0.03–0.21 (Figure 5).

## Discussion

With the continuous advancements in critical care and therapeutic strategies, the survival rate of infants diagnosed with intestinal atresia has shown a significant improvement, exceeding 90% (13, 14). In cases of non-complex intestinal atresia, primary anastomosis has been widely adopted by surgeons due to its effectiveness in reducing complications associated with ostomy formation (3, 4, 15). However, postoperative complications, including adhesive intestinal obstruction, anastomotic leakage, and stricture, frequently necessitate reoperation (4–6), particularly unplanned reoperations within the 30-day postoperative period. These complications significantly influence postoperative recovery and the overall health outcomes of affected infants, thereby establishing unplanned reoperation rates as a crucial indicator for assessing healthcare quality. In recent years, this issue has attracted growing attention in both clinical research and practice, highlighting the need for further investigation and optimization of surgical management strategies (16–18).

Cui et al. (9) reported that the incidence of unplanned reoperation in neonates undergoing primary repair of gastrointestinal disorders was 9.8% (29/296), among which 89.7% (26/29) of the cases involved patients who underwent enterectomy. The study revealed that patients suffering from enterectomy were more prone to requiring unplanned reoperation. Owing to the significant disparity in the diameter of the proximal and distal intestinal segments in cases of intestinal atresia, as well as the frequent association with MP, the rate of reoperation in these cases is notably higher compared to general intestinal surgeries.

This study identified that intestinal atresia complicated by MP is a significant risk factor for unplanned reoperation, with the probability of unplanned reoperation being 3.29 times higher compared to cases of isolated intestinal atresia. Nonetheless, the contrasting findings proposed by Chan et al. (19) demonstrates that the reoperation rates were comparable between patients with MP and those with isolated intestinal atresia (12% vs. 20%, *P* = 0.606), which reflects that the presence of MP has no correlation with the increase of reoperation risk.

Furthermore, this study highlights that the method of intestinal anastomosis is another critical risk factor for unplanned reoperation. Specifically, other anastomotic techniques (including end-to-end, side-to-side, and diamond-shaped anastomoses) were associated with a 6.34-fold higher risk of reoperation compared to end-to-oblique anastomosis. This finding underscores the superiority of the end-to-oblique anastomosis technique in the surgical management of intestinal atresia. Consistent with our results, Joda et al. reached the conclusion that end-to-side oblique anastomosis leads to a wide and early-functioning anastomosis, representing a straightforward but effective surgical procedure for intestinal atresia (20).

The classification of intestinal atresia significantly impacts the prognosis of affected infants. Since being considered as complex forms of intestinal atresia, it is reasonable to presume that Types IIIb and IV atresias are closely linked to a higher risk of postoperative complications and reoperation (7). Zhu et al. (21) claimed that the incidence of unplanned reoperation was 17.9% in neonates with apple-peel atresia. In our study, the risk of reoperation for type IV intestinal atresia was 3.17 times higher than that for type I, while the risks for types II and III were 1.61 and 1.91 times higher, respectively. However, further analysis revealed that the classification of intestinal atresia was not an independent risk factor for unplanned reoperations.

Certainly, the factors contributing to unplanned reoperations are not merely the intestinal anastomosis methods and whether their combination with MP obtained in this study. For instance, Yeung et al. noticed that prematurity and low birth weight were associated with functional obstruction leading to reoperation (5). Other studies have also identified long operation time (9), emergency surgery conducted at night (22), and surgical technical errors (18) as significant reasons for unplanned reoperation.

The establishment of a predictive model for the risk of reoperation after neonatal intestinal atresia surgery will visually present the relationship between postoperative reoperation-related risk factors and the likelihood of postoperative reoperation via a nomogram. This approach tends to be conducive for medical staff to paying attention to the potential risks of postoperative reoperation in children, conducting early interventions for high-risk patients, and choosing temporary enterostomy when necessary. Additionally, interdisciplinary integration for targeted intervention and risk management also promotes the initiative and foresight of postoperative nursing work, thereby helping to reduce the incidence rate of postoperative reoperation in patients. Furthermore, it offers early psychological preparation for both medical staff and family members of affected children to alleviate doctor-patient disputes.

This study has several limitations. First, This study is a single-center retrospective study, which needs to be further confirmed by future studies. Second, patients' individual differences and comorbidities may have potential effects on the reoperation rate. Third, due to variations in treatment and nursing care, it is unclear whether our results are applicable to other centers.

## Conclusion

To the best of our knowledge, this is the first report about establish a nomogram to predict thirty-day unplanned reoperations of primary anastomosis in neonates with IA. This study identified that type of bowel anastomosis and combined with MP were the risk factors of unplanned reoperation. A straightforward risk prediction model that includes these factors can assist in clinical decision-making, patient counseling, and treatment planning.

## Data Availability

The raw data supporting the conclusions of this article will be made available by the authors, without undue reservation.
